# Hemicentin-1 is an essential extracellular matrix component during tooth root formation by promoting mesenchymal cells differentiation

**DOI:** 10.3389/fcell.2024.1435241

**Published:** 2024-07-10

**Authors:** Yujia Cui, Chuwen Li, Hanyang Wang, Lei Li, Jing Xie, Xuedong Zhou, Hai Zhang, Jianxun Sun

**Affiliations:** ^1^ State Key Laboratory of Oral Diseases and National Center for Stomatology and National Clinical Research Center for Oral Diseases, Department of Pediatric Dentistry, West China Hospital of Stomatology, Sichuan University, Chengdu, China; ^2^ State Key Laboratory of Oral Diseases and National Center for Stomatology and National Clinical Research Center for Oral Diseases, Department of Cariology and Endodontics, West China Hospital of Stomatology, Sichuan University, Chengdu, China; ^3^ Shanghai Key Laboratory of Stomatology and Shanghai Research Institute of Stomatology and National Clinical Research Center for Oral Diseases and Shanghai Ninth People’s Hospital, College of Stomatology, Shanghai Jiao Tong University School of Medicine, Shanghai, China; ^4^ State Key Laboratory of Oral Diseases and National Center for Stomatology and National Clinical Research Center for Oral Diseases, Department of Prosthodontics, West China Hospital of Stomatology, Sichuan University, Chengdu, China; ^5^ State Key Laboratory of Oral Diseases and National Clinical Research Center for Oral Diseases, West China Hospital of Stomatology, Sichuan University, Chengdu, China; ^6^ School of Dentistry, University of Washington, Seattle, WA, United States

**Keywords:** dental pulp, dentinogenesis, extracellular matrix, hemicentin-1, microarray analysis, tooth root

## Abstract

**Introduction:** Root dentin formation is an important process in tooth development. We tried to identify potential genes that regulate root dentin formation which could be potentially used for the regeneration and repair of defective or damaged dental roots.

**Methods:** Tissues harvested from the labial and lingual sides of mouse incisors were used for microarray analysis. Gene ontology (GO) analysis of differentially expressed genes indicated the critical role of extracellular matrix in the discrepancy of dentin formation between root and crown, for which hemicentin-1 (*Hmcn1*) was selected as the target gene. Single-cell RNA sequencing analysis the expression pattern of *Hmcn1* at different developmental stages in mouse molars. The spatiotemporal expression of *HMCN1* in mouse incisors and molars was detected by immunohistochemical staining. The functions of HMCN1 in human dental pulp cells, including proliferation, differentiation and migration, were examined in vitro by CCK8 assay, BrdU assay, wound-healing assay, ALP staining and alizarin red staining, respectively.

**Results:** It was showed that *HMCN1* expression was more pronounced in papilla-pulp on the root than crown side in mouse incisors and molars. *In vitro* experiments presented inhibited dentinogenesis and migration after *HMCN1*-knockdown in human dental pulp cells, while there was no significant difference in proliferation between the *HMCN1*-knockdown group and control group.

**Discussion:** These results indicated that *HMCN1* plays an important role in dentinogenesis and migration of pulp cells, contributing to root dentin formation.

## 1 Introduction

The formation of dental organs is accomplished through a series of coordinated molecular interactions between the epithelial and mesenchymal cell populations of the tooth, beginning with the development of the crown and ending with the formation of the root. Previous studies have identified several important genes that are important in crown dentin formation (Thesleff and Sharpe, 1997; Jernvall and Thesleff, 2000; Tucker and Sharpe, 2004; Zhang, et al., 2005) but few in root dentin formation (Bosshardt and Nanci, 1997; Huang, et al., 2010; Liu et al., 2015; Huang X. et al., 2018). The root dentin is very important in clinical practice since the root supports the whole tooth, and short or thin root dentin can be a great challenge to root canal therapy. A thorough understanding of the precise molecular mechanisms of root dentin formation is not only crucial to tooth repair and regeneration, but also provides new ideas for clinical treatment.

Extracellular matrix (ECM) is a crucial component of the stem cell microenvironment and contributes to the regulation of mesenchymal cells differentiation to form dental root dentin. ECM regulates the transition of stem cells from quiescence to readiness to sense differentiation-inducing signals and the transition between different stages of differentiation (Novoseletskaya, et al., 2023). Besides, ECM proteins can activate intracellular signalling pathways such as the extracellular signal-regulated kinase pathway or the focal adhesion kinase pathway to induce osteogenic differentiation of mesenchymal stem cells (Morsczeck, 2022). During tooth morphogenesis, ECM whose main components are laminin, collagen IV, nidogen and sulfated proteoglycan are dramatically changed (Yoshizaki and Yamada, 2013). Therefore, accurate knowledge of the function of ECM proteins is important for understanding molecular mechanisms of root dentin formation.

Hemicentin-1 (HMCN1) is an evolutionarily highly conserved ECM protein that is also called fibulin-6, a member of the fibulin family of secreted glycoproteins (de Vega, et al., 2009; Jordan, et al., 2011). This ECM protein was first found as an analog in *Caenorhabditis elegans* called hemicentin, which plays an important role in cell migration and cell-cell or cell-basement conjunction formation (Vogel and Hedgecock, 2001; Bonacossa-Pereira et al., 2022). Also, in mammals, HMCN1 is essential in the dermal–epidermal and myotendinous junctions (Welcker et al., 2021). Besides, previous studies have demonstrated that HMCN1 could modulate transforming growth factor-β (TGF-β) activation and affect its signal transduction. It has been proven that HMCN1 is involved in the process of TGF-β signaling regulating the formation of fibers in neonatal mouse ventricular cardiac fibroblasts by influencing the interaction of TGF-β receptors I and II (Chowdhury et al., 2014; Chowdhury et al., 2017). Interestingly, recent work on tissue regeneration of planarians indicated that HMCN1 was required for maintaining parenchymal neoblast and parenchymal differentiated cell localization, which revealed the critical role of HMCN1 in cell location information in development (Cote, et al., 2019). However, it remained unknown whether HMCN1 participated in root dentin formation.

The mouse incisor is a lifelong growing, asymmetrical organ that continually forms enamel (crown) on the labial side and cementum (root) on the lingual side (Yu and Klein, 2020). This organ is an excellent tool for studying the differences between the root and crown of the teeth. Here, we used mouse incisors to identify important genes that have unique functions in dental root formation by comparing different expression levels of gene transcripts between the labial and lingual sides, and *Hmcn1* was identified as a potential regulator of root dentin formation. Then, to examine the potential role of hemicentin-1 in root dentin formation, we analyzed the expression of *Hmcn1* in single-cell RNA sequencing datasets of different mouse molar developmental stages. We further identified the localization and distribution of HMCN1 in mouse incisors and molars. Lastly, we utilized dental pulp cells as a type of mesenchymal cell, which is involved in root dentin formation, to explore the functions of HMCN1 *in vitro*.

## 2 Materials and methods

### 2.1 Laser capture microdissection and microarray analysis

The animal experiment was performed following the protocols approved by the Ethics Committee of West China School of Stomatology, Chengdu, China (SKLODLL 2013A035).

Tissues were prepared and processed as described in a previous study (Sun et al., 2012). Briefly, the sagittal sections of the mouse mandibles from three CD-1 mice on P7 were fixed with 95% ethanol for 1 min, stained with 2.8% cresol violet dissolved in 75% ethanol, and then washed in 95% ethanol twice for 1 min each time. After that, the section was washed with 100% ethanol twice. After dye treatment, the polyethylene naphtha membrane loading slide was placed in a laser capture microdissection workstation for specific tissue collection. Six groups of tissues—the cervical loop, pulp-papilla, and dental follicle from both the labial and lingual sides were harvested for RNA extraction. RNA was extracted from the collected tissue with an RNA extraction kit (PicoPure, Arcturus Bioscience, United States), and the RNA with a high RNA integrity number (RIN) was used for microarray analysis (Gene Chip Scanner 3000, Affymetrix, United States).

### 2.2 Principal component analysis (PCA)

The above data were used for PCA of microarray analysis Min-max normalization was utilized to normalize the selected data and the data were centered with the mean subtraction approach. OriginPro 2017 software (OriginLab, MA, United States) was used to get PCA for microarray analysis. PC1, PC2, and PC3 were utilized to represent the location of each element in a 3D graph.

### 2.3 Gene functional enrichment analysis

The transcriptome profile of the samples from microarray analysis was used for Gene Ontology (GO) functional annotation analysis (http://geneontology.org/) and ranked by 3 different algorithms: classic, elim and weight. Several genes that had significantly different expression between the labial and lingual sides (fold change, FC ≥ 2 and p-value < 0.05) were compared with the GO analysis results of the transcriptome profile. Three independent ontologies, including the biological process (BP), molecular function (MF), and cellular component (CC) categories were constructed to describe gene product attributes.

### 2.4 Re-analysis of single-cell RNA sequencing dataset

The single-cell RNA sequencing data was from the Gene Expression Omnibus (GEO) dataset GSE189381, deposited by Jing J (Jing et al., 2022). Single-cell transcriptomes were obtained from the digestion of the tooth and its surrounding tissue at different stages (E13.5, E14.5, E16.5, PN3.5, and PN7.5) of mouse moalrs. To analyze the raw read counts at each stage, we utilized the R package Seurat (Ver.4.0) for downstream single-cell level analyses. Cells expressing genes with fewer than 200 genes and mitochondrial genes constituting more than 20% of the expression were filtered out. “SCTransform” was applied for normalization and cell cycle regression. RunPCA and RunUMAP were performed for dimensionality reduction and final visualization of the clustering.

### 2.5 Tissue fixation and immunohistochemical (IHC) staining

Mice were euthanized at 1, 7, 10, 14, and 21 days after birth, and the mouse heads were soaked in Bouin’s fixation solution for 24 h, washed for 3 times with 70% ethanol, and preserved in 70% ethanol at 4°C. These heads were then dissected to harvest the mandibles (samples taken 1 day after birth were treated without dissection), which were cut in half before they were placed in an AFS demineralization solution for 1∼3 weeks at 4°C (no demineralization for the samples from 1-day-old mouse pups). Dehydrated samples embedded with paraffin were set horizontally, fixed at room temperature, and sectioned.

The immunohistichemical staining was performed as in our previous study (Cui et al., 2023). After deparaffinization and hydration of the tissue slices, antigen repair was conducted using citrate antigen re-trieval solution (Beyotime, Shanghai, China) at 100°C for 15 min. Next, the tissue slices were treated with H_2_O_2_ for 30 min and blocked for 1 h. Then, tissue sections were treated with an immunohistochemical staining kit (Vector, United States) and stained with an anti-mouse hemicentin-1 antibody (ProteinTech, United States) followed by horseradish peroxidase staining (AEC horseradish peroxidase staining kit, Vector, United States), and were finally stained with Harris’ hematoxylin (EMD company, United States).

### 2.6 Isolation and culture of dental pulp cells

Human dental pulp was extracted from integrated third molars without caries from healthy adults aged 18–20 in the West China Hospital of Stomatology under a protocol approved by the hospital’s Ethics Committee (WCHSIRB-D-2021-129). The dental pulps were digested for 2–4 h by collagenase type I (3 mg/mL, dissolved in PBS, Gibco, Life Technologies) to isolate human dental pulp cells (HDPCs). After centrifugation, the pelleted cells were maintained in 6 cm dishes in the common medium consisting of Dulbecco’s modified Eagle’s medium (DMEM) containing high glucose (4.5 mg/mL) and supplemented with 10% fetal bovine serum (FBS) and 1% penicillin-streptomycin (PS) mixture. These cells were passaged once or twice in 10-cm dishes before transfection.

### 2.7 ShRNA transfection

Cells were seeded into 6-well plates and incubated for 12 h before transfection. Lentiviral vectors containing *HMCN1*-knockdown shRNA or scramble control as well as the GFP and PURO genes were synthesized by Hanheng Biological Engineer Company (Shanghai, China). The HMCN1-knockdown and scramble vectors with polybrene (3 μg/mL) were added to 30%–40% confluent HDPCs. The medium was changed to the common medium 18 h later and changed again to medium with 2 μg/mL puromycin when fluorescence was observed (approximately 72 h later). Forty-8 hours later, the cells were maintained in the common medium for the following experiments.

### 2.8 Mineralization and differentiation induction

The mineralization induction was performed as in our previous study (Cui et al., 2020). Post-transfected dental pulp cells were maintained in a mineralization-induced medium, which consisted of DMEM, 10% FBS, 1% PS, 10 mmol/L β-sodium glycerophosphate, 50 mg/L vitamin C and 0.1 μ mol/L dexamethasone (Sigma-Aldrich, United States). Briefly, cells were treated with mineralization-induced medium for 7 days. The medium was changed every 2 days.

### 2.9 ALP and alizarin red staining

Post-transfected dental pulp cells treated with mineralization-induced medium for 7 days in 24-well plates were fixed with 4% polyoxymethylene for 10 min and washed 3 times with PBS. Then ALP (Beyotime, China) and alizarin red (Cyagen, China) staining was performed following their standard protocols.

### 2.10 RT-qPCR

Total RNA was extracted from cells with Trizol reagent (Invitrogen, United States) following the standard protocol. Reverse transcription of the total RNA was conducted with the PrimeScript™ RT reagent Kit (TaKaRa, Japan). Then, RT-qPCR was performed with TB Green™ Premix Ex Taq™ II (TaKaRa, Japan) with a CFX96 Real-Time System (Bio-Rad). The mRNA expressed by target genes was quantified using the Cq results, using *GAPDH* as an internal control. The specifc primer sequences used for RT-qPCR are listed in Table 1. Three replicates were made for each group. The statistical analysis of RT-qPCR was performed in the software GraphPad Prism 7.

**TABLE 1:** Primer sequences used in RT-qPCR.

Gene	Primer sequences (5′–3′)
*DSPP*	ATA​TTG​AGG​GCT​GGA​ATG​GGG​A (Forward)
	TTT​GTG​GCT​CCA​GCA​TTG​TCA (Reverse)
*GAPDH*	GGA​GCG​AGA​TCC​CTC​CAA​AAT (Forward)
	GGC​TGT​TGT​CAT​ACT​TCT​CAT​GG (Reverse)
*HMCN1*	TCT​TGC​GCT​GGA​TTG​GAG​AG (Forward)
	CAG​AGC​CTT​CAG​GGC​AAG​TT (Reverse)
*RUNX2*	CCT​TTA​CTT​ACA​CCC​CGC​CA (Forward)
	GGA​TCC​TGA​CGA​AGT​GCC​AT (Reverse)
*OSX*	TCT​GCG​GGA​CTC​AAC​AAC​TC (Forward)
	TAG​CAT​AGC​CTG​AGG​TGG​GT (Reverse)
*Hmcn1*	ACC​TGT​GAT​CAT​GTG​GCT​CA (Forward)
	GGA​TTC​TTA​CTC​GAG​GTG​TTG​C (Reverse)
*Gapdh*	TCC​ACC​ACC​CTG​TTG​CTG​TA (Forward)
	ACC​ACA​GTC​CAT​GCC​ATC​AC (Reverse)

### 2.11 Proliferation assay

Cell proliferation was tested using a Cell Counting Kit-8 (CCK-8) from Dojindo (Shanghai, China) according to the manufacturer’s protocol. The 5-bromo-2′-deoxyuridine (BrdU) cell proliferation assay was carried out with a BrdU Cell Proliferation Assay Kit (BioVision, United States) following the manufacturer’s protocol. The OD values of the CCK-8 and BrdU proliferation assay were measured with a spectrophotometer (Thermo Fisher Science Oy Ratastie 2, FI-01620 Vantaa, Finland). The statistical analysis of CCK8 and BrdU proliferation assay was performed in the software GraphPad Prism 7.

### 2.12 Wound-healing assay

The experimental process of the wound-healing assay was performed as described in a previous report (Grada, et al., 2017). Post-transfected dental pulp cells were cultured in 6-well plates in DMEM supplemented with 10% FBS and 1% PS until a fully confluent monolayer was formed. A sterile plastic micropipette tip was then used to create a straight-edged, cell-free wound across the cell monolayer in each well. The culture medium was changed to serum-free DMEM. The pictures of the wound areas were taken at 0 and 24 h after scratching, and the areas were calculated by ImageJ. The statistical analysis of wound-healing assay was performed in the software GraphPad Prism 7.

### 2.13 Statistical analysis

The results are presented as the mean ± S.E.M. of at least three individual experiments. The data were analyzed by one-way ANOVA followed by Tukey’s protected least-significant difference *post hoc* test for multiple comparisons. *p* < 0.05 indicated a statistically significant difference.

## 3 Results

### 3.1 Incisor tissues from the lingual and labial sides exhibited significant differences in terms of extracellular matrix

**FIGURE 1:**
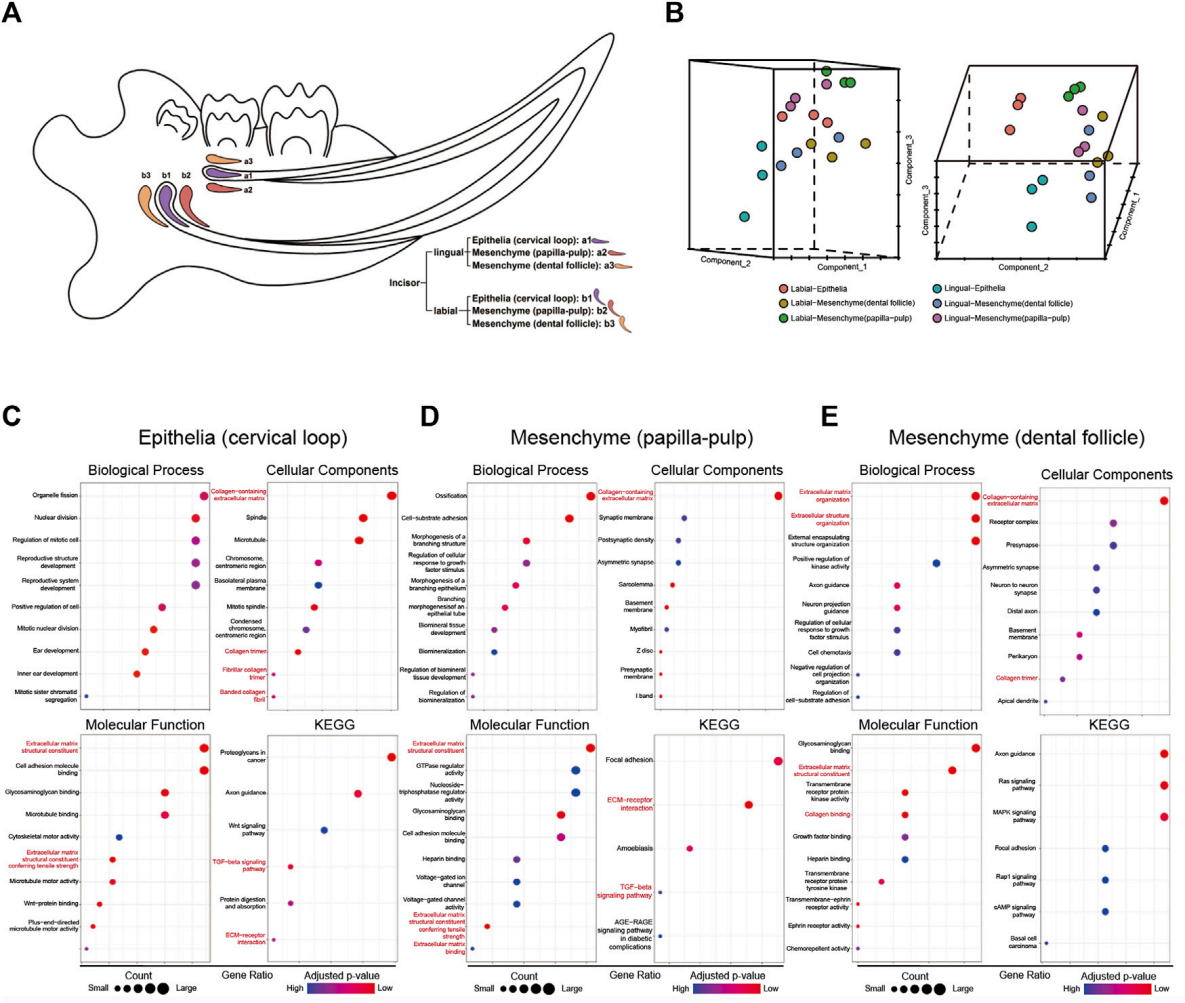
The microarray results of tissues from mouse incisors obtained by laser capture microdissection. **(A)** The sketch map of microdissection. The following 6 cell populations were captured: incisor-lingual (future root): a1. epithelia (cervical loop), a2. mesenchyme (papilla-pulp) and a3. mesenchyme (dental follicle); incisor-labial (future crown): b1. Epithelia (cervical loop), b2. mesenchyme (papilla-pulp) and b3. mesenchyme (dental follicle). **(B)** PCA analysis of microarray results for samples from microdissection. **(C–E)** Enrichment analysis of differential expression genes for comparisons of lingual and labial tissues from cervical loop, papilla-pulp and dental follicle, respectively. Extracellular matrix-related entries were red-labelled.

To identify critical signaling factors in root dentin formation, different incisor tissues from the labial side and the lingual side were dissected by laser capture microdissection (Figure 1A). RNA extracted from these tissues was used for microarray analysis. The microarray analysis presented different expression patterns of the transcriptomes between the labial and lingual sides of mouse incisors (Figure 1B). The GO functional annotation analysis of the transcriptomes suggested “extracellular region” as one of the most important entries involved in root dentin formation compared with crown dentin formation (Table 2). It was also noticeable that ECM-related entries, such as “ECM structural constituent,” “ECM-receptor interaction” and “ECM binding,” were frequently annotated in the enrichment analysis of differentially expressed genes in the three comparisons (Figures 1C–E). These results indicated that ECM was one of the critical factors in the discrepancy between root dentin and crown dentin formation.

**TABLE 2:** The GO entries with top 20 ranking in all the samples from the GO analysis.

GO	Sample	GO coding	GO entry	Rank		
				Classic	Elim	Weight
BP	Cervical loop	GO:0051301	cell division	20	2	2
		GO:0007275	multicellular organismal development	1	13	10
	Pulp-papilla	GO:0007155	cell adhesion	7	1	1
	Dental follicle	GO:0007155	cell adhesion	6	1	1
CC	Cervical loop	GO:0005737	cytoplasm	13	3	2
		GO:0005886	plasma membrane	19	7	5
		GO:0005576	extracellular region	2	9	8
	Pulp-papilla	GO:0005576	extracellular region	1	1	1
		GO:0042383	sarcolemma	10	4	3
		GO:0005886	plasma membrane	7	6	13
	Dental follicle	GO:0005576	extracellular region	1	2	1
		GO:0043209	myelin sheath	16	14	3
		GO:0009986	cell surface	20	10	4
MF	Cervical loop	GO:0005509	calcium ion binding	4	2	2
		GO:0005539	glycosaminoglycan binding	8	20	3
		GO:0005524	ATP binding	10	3	4
	Pulp-papilla	GO:0046872	metal ion binding	3	4	1
		GO:0070700	BMP receptor binding	12	2	3
		GO:0005515	protein binding	2	17	10
	Dental follicle	GO:0005515	protein binding	2	9	2
		GO:0046872	metal ion binding	6	7	3
		GO:0017153	sodium: dicarboxylate symporter activity	18	4	5

### 3.2 *Hmcn1* had a higher expression on the lingual side in cervical loop and pulp-papilla

**FIGURE 2:**
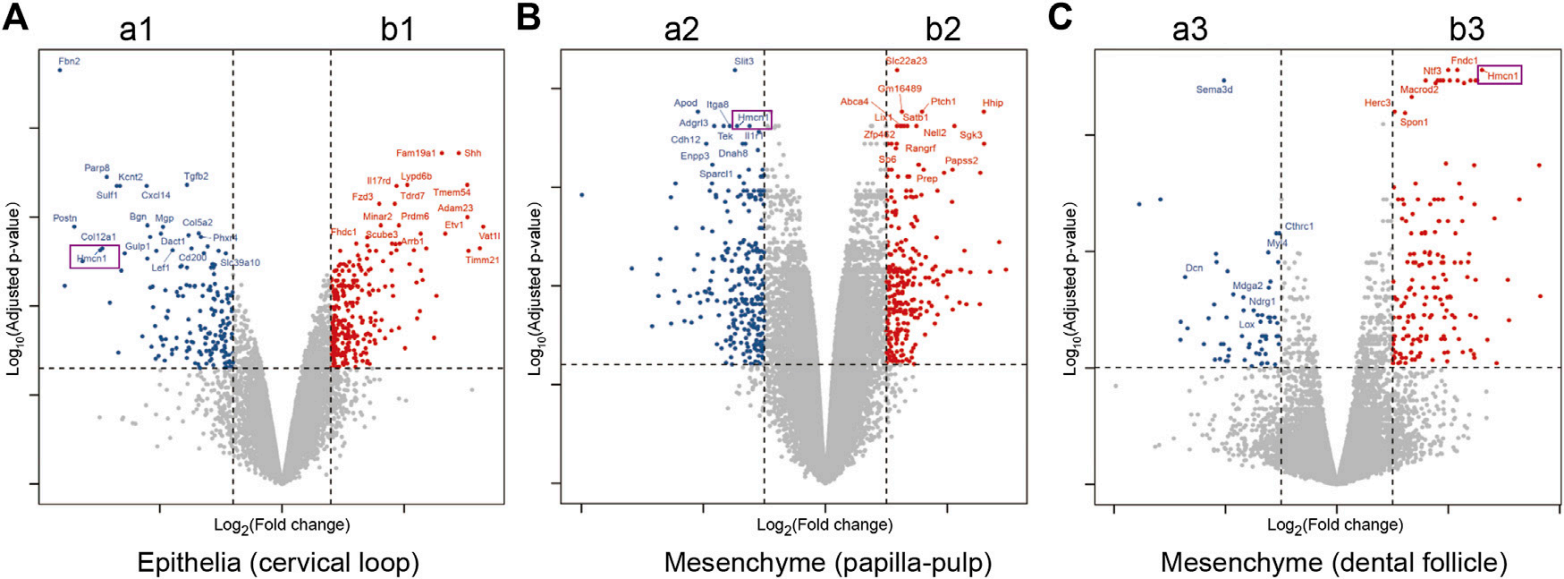
Differential expression genes for comparisons of lingual and labial tissues from cervical loop, pulp-papilla and dental follicle. **(A)** Volcano plots for the comparison of lingual and labial tissues from cervical loop. **(B)** Volcano plots for the comparison of lingual and labial tissues from papilla-pulp. **(C)** Volcano plots for the comparison of lingual and labial tissues from dental follicle. Hmcn1 was labelled by purple rectangles.

To identify critical genes involved in root dentin formation, differentially expressed genes were further investigated. Among the genes with significantly differential expression, 9 genes were ranked at the top of the fold-change list between the labial and lingual sides, namely, *Nmnat2, Hmcn1, Slit3, Fam20a, Rspo4, Pdlim5, A930038C07Rik, C1s* and *Pdgfd* (Table 3). We noticed that *Hmcn1*, which codes for an ECM protein with a fibulin-like domain, presented significantly differential expression levels between labial and lingual sides in all three comparisons (Figures 2A–C). Since epithelia and pulp-papilla on the lingual side are directly contribute to root dentin formation when the dental follicles do not, genes involved in the root dentin formation could be upregulated in the two former tissues but might be irrelevant with the expression in the dental follicle. Interestingly, both the cervical loop and pulp-papilla expressed higher levels of *Hmcn1* on the lingual side, whereas the dental follicle exhibited a higher level of *Hmcn1* on the labial side. Thus, we hypothesized that the ECM protein HMCN1 could be one of the key regulators for root dentin formation.

**TABLE 3:** Nine genes with significantly different expression levels in all three tissues evaluated.

Gene name	Cervical loop		Pulp-papilla		Dental follicle	
	log_2_ FC	p-value	log_2_ FC	p-value	log_2_ FC	p-value
*Nmnat2*	−1.61996	0.005261	1.88018	0.001803	−1.94197	0.0014
*Hmcn1*	−3.14024	0.00032	−1.34863	0.015045	1.917632	0.002663
*Slit3*	−1.52524	9.55E-06	−1.46374	1.51E-05	1.042657	0.000449
*Fam20a*	−1.13337	0.013392	1.063436	0.018938	−1.44951	0.002723
*Rspo4*	−1.29632	0.03709	−1.47092	0.020283	1.476923	0.019861
*Pdlim5*	−2.44573	3.36E-05	−1.49024	0.002883	−1.06418	0.022641
*A930038C07Rik*	−1.43518	0.002899	−1.16813	0.01117	−1.4184	0.003156
*C1s*	−1.51512	0.000642	−1.47433	0.00081	1.027797	0.010679
*Pdgfd*	−1.06954	0.012821	−1.01988	0.016684	−1.29887	0.003736

Note 1: The screening conditions were log2. FC ≥ 1 and p-value<0.05.

Note 2: When log2 FC, is positive, the gene is expressed at a high level on the labial (crown) side; when log2 FC, is negative, the gene is expressed at a high level on the lingual (root) side.

Note 3: *Hmcn1* has multiple transcripts, the average of which is listed in the table.

### 3.3 Spatiotemporal expression of HMCN1 in incisors and molars of mice

**FIGURE 3:**
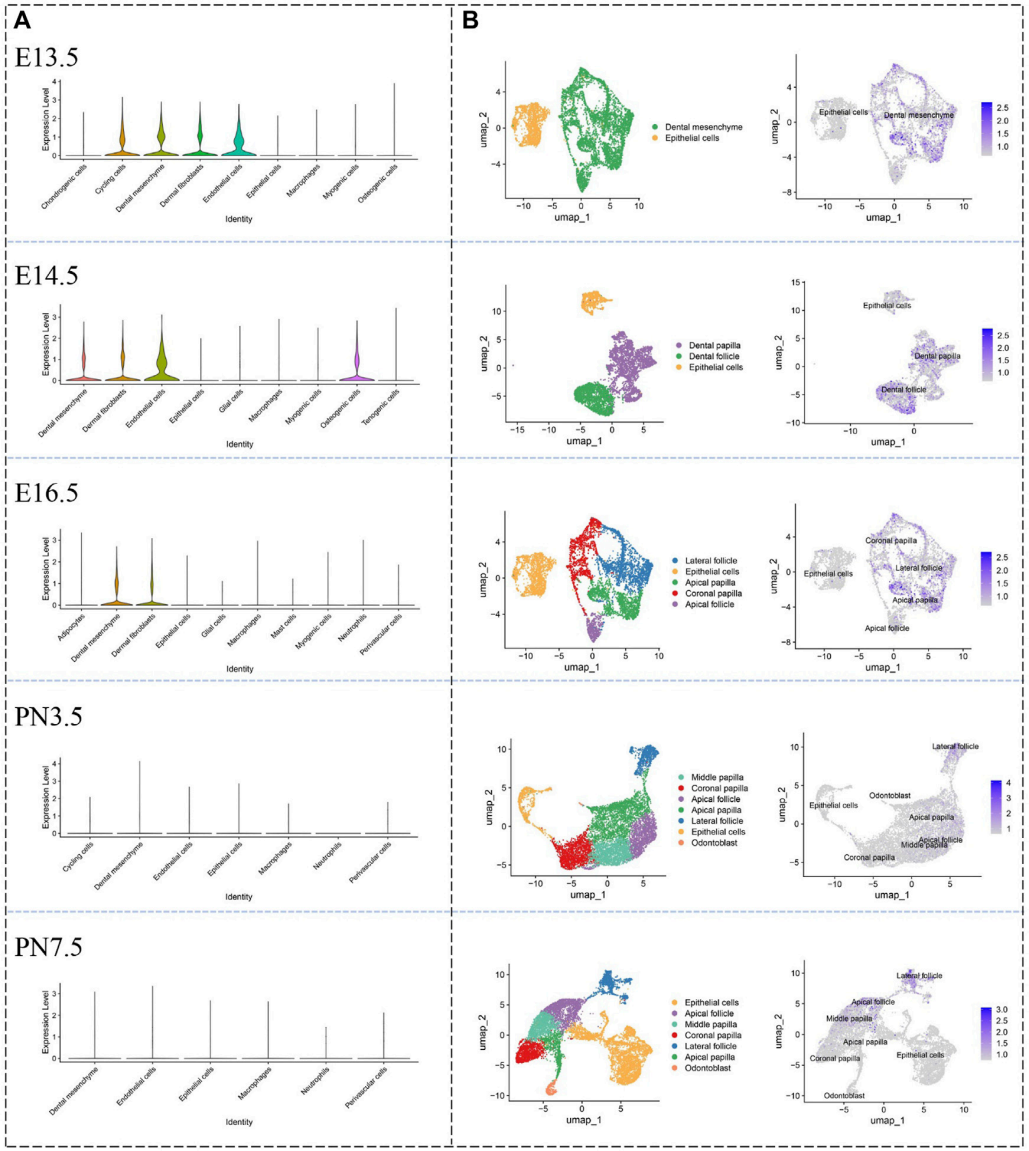
Defining expression pattern of Hmcn1 at different developmental stages in the single-cell transcriptomic atlase. **(A)** Violin plots of the expression of Hmcn1 in distinct clusters at different stages (E13.5, E14.5, E16.5, PN3.5 and PN7.5) of mouse moalrs. **(B)** UMAP visualization of Hmcn1 expression in dental mesenchyme subclusters at E13.5, E14.5, E16.5, PN3.5 and PN7.5.

Violin plots (Figure 3A), which was used to visually represent the single-cell data from E13.5 to postnatal (PN) 7.5, showed *Hmcn1* highly expressed in cycling cells, dental mesenchyme, dermal fibroblasts and endothelial cells, whereas it was little expressed in chondrogenic cells, epithelial cells, macrophages and myogenic cells and osteogenic cells at E13.5. At E14.5, *Hmcn1* highly expressed in dental mesenchyme, dermal fibroblasts, endothelial cells and osteogenic cells, whereas it was little expressed in epithelial cells, glial cells, macrophages, myogenic cells and tenogenic cells. At E16.5, *Hmcn1* highly expressed in dental mesenchyme and dermal fibroblasts, whereas it was little expressed in adipocytes, epithelial cells, glial cells, macrophages, mast cells, myogenic cells neutrophils and perivascular cells. At PN3.5 and PN7.5, there was no difference in the expression of *Hmcn1* between different clusters. UMAP plots (Figure 3B) further exhibited the expression of *Hmcn1* in the dental mesenchyme subclusters. Since E14.5, dental follicle and dental papilla segregated. *Hmcn1* expressed in dental papilla and dental follicle at E14.5. *Hmcn1* expressed highly in apical papilla, coronal follicle and lateral follicle at E16.5. At PN3.5 and PN7.5, dental papilla formed separate subclusters, including coronal papilla, middle papilla, apical papilla and odontoblast. At PN3.5, *Hmcn1* expressed in apical papilla, middle papilla, apical follicle and lateral follicle. At PN7.5, *Hmcn1* expressed in middle papilla, apical follicle and lateral follicle.

**FIGURE 4:**
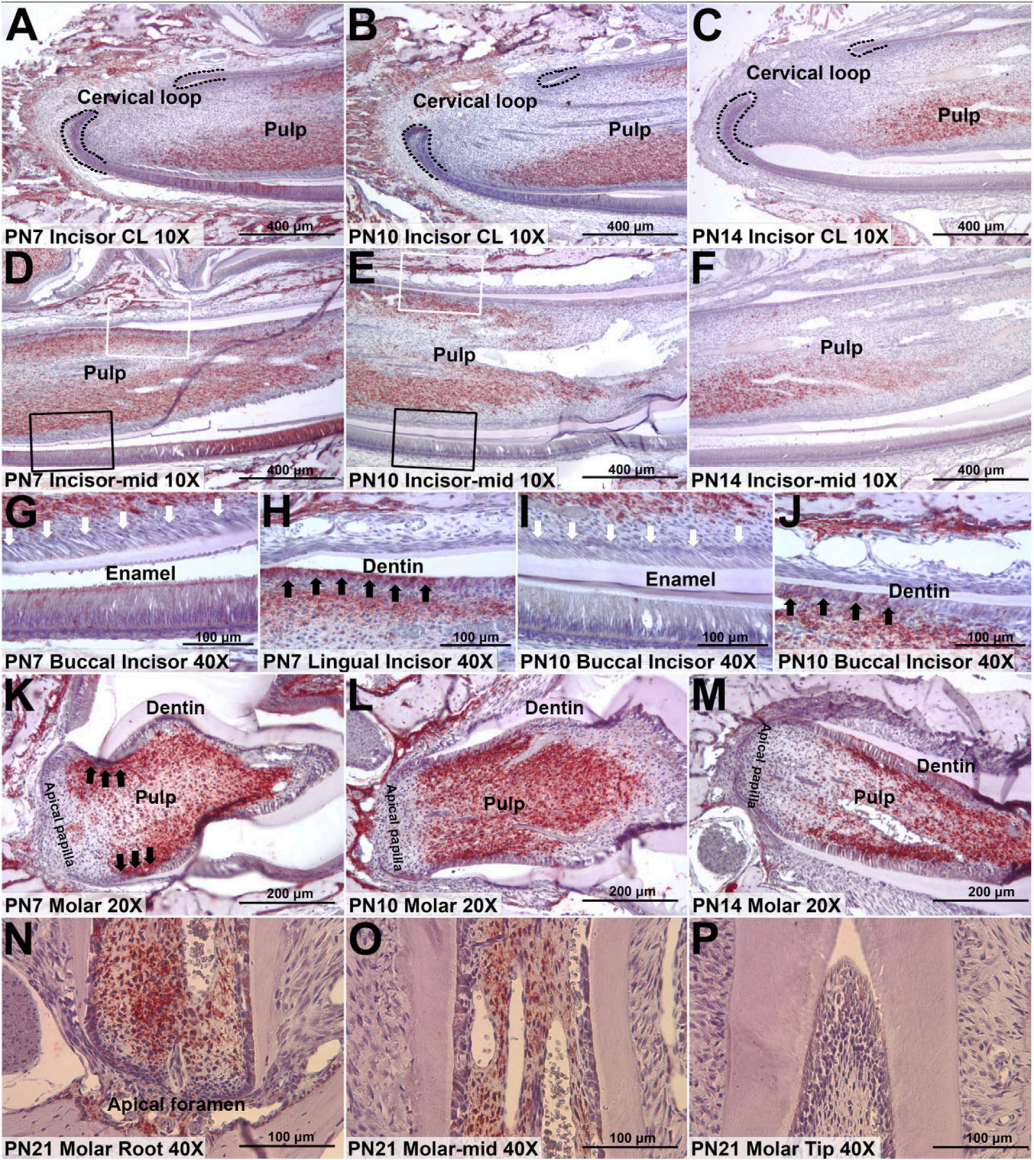
Representative IHC staining of HMCN1 in incisors and molars of mice. **(A)** The expression of HMCN1 in cervical loop of PN7 mouse incisors. **(B)** The expression of HMCN1 in cervical loop of PN10 mouse incisors. **(C)** The expression of HMCN1 in cervical loop of PN14 mouse incisors. **(D)** The expression of HMCN1 in the middle part of PN7 mouse incisors. **(E)** The expression of HMCN1 in the middle part of PN10 mouse incisors. **(F)** The expression of HMCN1 in the middle part of PN14 mouse incisors. **(G)** Enlarged images of labial side marked by black rectangles in **(D)**. White arrowheads indicate the lack of HMCN1. **(H)** Enlarged images of lingual side marked by white rectangles in **(D)**. Black arrowheads indicate HMCN1 expression. **(I)** Enlarged images of labial side marked by black rectangles in **(E)**. White arrowheads indicate the lack of HMCN1. **(J)** Enlarged images of lingual side marked by white rectangles in **(E)**. Black arrowheads indicate HMCN1 expression. **(K)** The expression of HMCN1 in sagittal sections of the first molars of PN7 mice. **(L)** The expression of HMCN1 in sagittal sections of the first molars of PN10 mice. **(M)** The expression of HMCN1 in sagittal sections of the first molars of PN14 mice. **(N)** The expression of HMCN1 in the root part of the first mandibular molar from PN21 mice. **(O)** The expression of HMCN1 in the middle part of the first mandibular molar from PN21 mice. **(P)** The expression of HMCN1 in the tip part of the first mandibular molar from PN21 mice.

To determine the protein expression location of HMCN1, IHC staining was conducted in the incisors and the first molars of PN mice. The IHC results indicated that the expression of HMCN1 was generally low in PN1 mice, with only part of the epithelial tissues around the incisor cervical loop expressing hemicentin-1 (Supplementary Figure S1). However, HMCN1 expression peaked in incisors and molars of PN7 mice and presented in the middle layer of the dental pulp and the odontoblasts on the lingual (root) side (Figure 4A). Consistent with the microarray results in mouse incisors, HMCN1 was expressed in pulp cells, lingual (root) odontoblasts and ameloblasts but showed little expression in cementoblasts or labial (crown) odontoblasts (Figures 4A–J). In the mandibular first molar, HMCN1 expression was concentrated on the root side of dental pulp as well, and the cells close to the outer layer of the dental pulp expressed more HMCN1(Figures 4K–M). The expression of HMCN1 showed the same distribution pattern at PN10 and PN14 as at PN7 but was decreased generally and more focused on lingual (root-side) odontoblasts. In addition, HMCN1 was still present on the root side of mandibular molars of PN21 (Figures 4N–P). These distribution patterns indicated the potential relationship between HMCN1 and root dentin formation.

### 3.4 HMCN1 promotes the differentiation and migration of dental pulp cells

**FIGURE 5:**
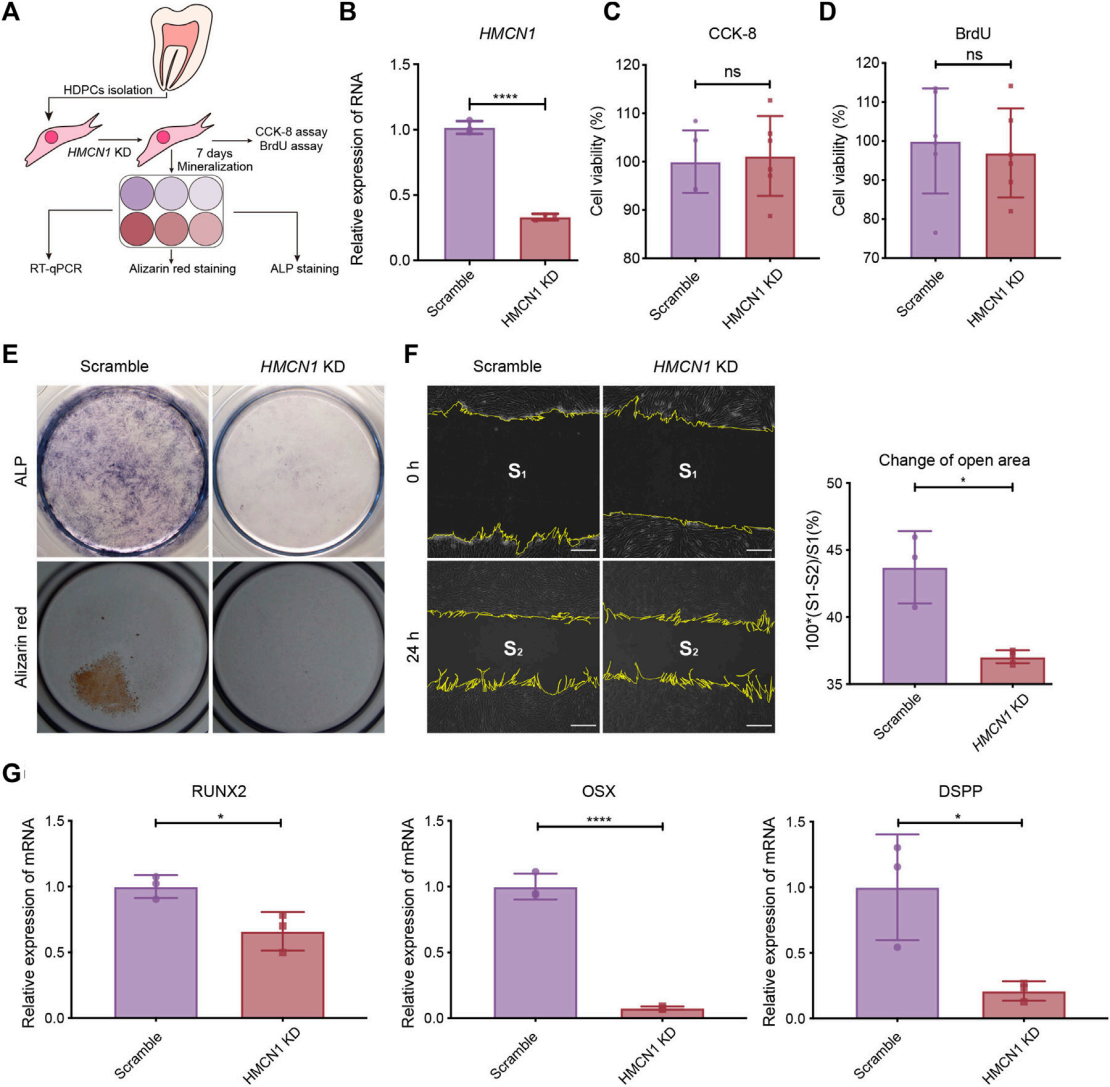
The influence of HMCN1 on the differentiation and migration process of hDPCs. **(A)** An introductory diagram showing the experimental steps. **(B)** The RT-qPCR results of HMCN1 of hDPCs transfected with lentivirus of HMCN1-knockdown shRNA. The bars represent the mean ± SEM, n = 3, ****p < 0.0001. **(C)** CCK-8 analysis of hDPCs after HMCN1-knockdown. The bars represent the mean ± SEM, n = 6, ns = no significant. **(D)** BrdU cell proliferation assay of hDPCs after HMCN1-knockdown. The bars represent the mean ± SEM, n = 6, ns = no significant. **(E)** Representative ALP staining and alizarin red staining of hDPCs after HMCN1-knockdown with mineralization induction for 7 days. **(F)** The Scratch wound healing assay evaluating hDPCs migration during 24 h in serum-free medium with/without HMCN1-knockdown. The bars represent the mean ± SEM, n = 3, *p < 0.05. Scale bar, 125 μm. **(G)** The RT-qPCR results of dentinogenesis-related genes (RUNX2, OSX, DSPP) in hDPCs with HMCN1-knockdown. The bars represent the mean ± SEM, n = 3, ****p < 0.0001.

To confirm the biological function of *HMCN1* in mesenchymal cells, which is important in the dentin formation, we conducted experiments *in vitro*, including CCK8 assay, BrdU assay, wound-healing assay, ALP staining, and alizarin red staining, respectively (Figure 5A). The expression of *HMCN1* was knocked down successfully in human dental pulp cells (hDPCs) by shRNA via a lentivirus vector (Figure 5B). Cell proliferation assays, including the CCK-8 assay and the BrdU assay, were also utilized to analyze the influence of *HMCN1* on cell proliferation (Figures 5C, D). The results did not show any differences between the HMCN1 knockdown (*HMCN1* KD) groups and the control groups. However, hDPCs in *HMCN1* KD group showed defective mineralization compared to that in the control group (Figure 5E). A scratch assay was performed and revealed that *HMCN1* KD cells exhibited deficient migration ability (Figure 5F). Transfected cells treated with a mineralization-induced solution for 7 days were evaluated by qRT-PCR. The mRNA levels of runt-related transcription factor 2 (RUNX2), Osterix (OSX), and dentin sialophosphoprotein (DSPP) were all decreased in the *HMCN1* KD groups, which indicated that the differentiation process was blocked during mineralization (Figure 5G). In summary, these results indicated that *HMCN1* promoted the differentiation of hDPCs but barely had an impact on cell proliferation.

## 4 Discussion

Odontogenesis is a complicated process regulated by genetic information and environmental factors. As root dentin formation is a crucial part of odontogenesis, it is valuable to reveal the precise regulatory mechanism of root dentin formation, more specifically, the differentiation process of pulp stem cells in dental roots. There must be some unique factors involved in root dentin formation since the process of dentin formation in the dental root is different from that in the dental crown. Identification of these unique factors could be helpful in understanding the process of root dentin formation and the regeneration of dental roots in adults.

In our study, the microarray analysis presented different gene expression profiles between the labial and lingual tissues harvested from mouse incisors. This result indicated different molecular mechanisms of tooth formation on the labial (root) side vs. that on the lingual (crown) side. Nine genes were highlighted after sorting: *Hmcn1, Nmnat2, Slit3, Fam20a, Rspo4, Pdlim5, A930038C07Rik, C1s* and *Pdgfd*. Among these 9 genes, the FC of *Hmcn1* in the cervical loop was the highest, which indicated that this gene may play a different role in root dentin formation vs. in crown. The microarray results revealed that, on the lingual side, higher expression of *Hmcn1* was found in the cervical loop and pulp-papilla but a lower expression level was found in the dental follicle, which suggested the potential regulatory role of *Hmcn1* in dental root formation since root dentin formation is the consequence of epithelia-mesenchyme interaction (EMI) between the cervical loop and pulp-papilla in the mouse incisor. The single-cell of mouse molars showed that *Hmcn1* exhibited higher expression in the dental mesenchyme subclusters than the epithelial cell subcluster. At E14.5 and E16.5, *Hmcn1* mainly expressed in dental papilla and dental follicle simultaneously. While at PN3.5 and PN7.5, when crown-root transition occurred, the expression of *Hmcn1* appeared to be heterogeneous. At PN3.5, *Hmcn1* expressed in apical papilla, middle papilla, apical follicle and lateral follicle. At PN7.5, *Hmcn1* expressed in middle papilla, apical follicle and lateral follicle. These results showed Hmcn1, expressed heterogeneous in dental mesenchymal cells. Among the different genes analyzed in the microarray, SLIT3, FAM20A, RSPO4, and PDGFD may also play a role in tooth dentin formation in addition to HMCN1. SLIT3, a member of the extracellular matrix protein SLIT family, is a new bone coupling factor, which plays a regulatory role in promoting bone formation and inhibiting bone resorption in bone metabolic balance (Jiang et al., 2022). In our latest study, we identified SLIT3 as a novel regulator of odontogenic differentiation through the Akt/Wnt/β-catenin signaling pathway (unpublished). Family with sequence similarity 20, member A (FAM20A) is a pseudo-kinase in the secretory pathway and is essential for enamel formation in humans, but is dispensable for dentinogenesis (Li et al., 2019). A recent study has shown that the reduction of FAM20A in mutant deciduous pulp cells is associated with a variety of cellular defects, including delayed proliferation, attachment, spreading, and migration, as well as altered osteogenic and inflammatory responses (Sriwattanapong et al., 2024). R-spondin 4 (RSPO4) is an activator of canonical Wnt signaling and has a role in osteogenesis. Pemberton T J, et al. analyzed microarray gene expression in the developing mouse molar tooth (Pemberton et al., 2007). RSPO4 was specific to the developing tooth in the craniofacial region. However, the specific function of RSPO4 in tooth development has not been proven currently. PDGFD is a member of the platelet-derived growth factor family. PDGFD was Identified as the putative odontogenic potential-related genes using the RNA-seq technique in E11.5 and E14.5 tooth germ (Shin et al., 2021). However, the specific function of PDGFD in tooth development has not been proven currently.

*Hmcn1* was first identified as its homolog *him-4 in C. elegans* (*C. elegans*), encoding a highly conserved ECM protein named hemicentin, which is a mnemonic for both its “hemicentum” structural modules and frequent association with hemidesmosomes (Vogel and Hedgecock, 2001). Hemicentin-1 is also called fibulin-6 for the fibulin-type carboxy-terminal modules (FCs), and contains multiple structural domains, including the von Willebrand A (VWA) domain, the epidermal growth factor (EGF) domain, and repeat immunoglobin domains. In previous studies, hemicentin-1 has been consistently found to be abnormal in several diseases, including Sjogren syndrome (Sisto et al., 2009), age-related macular degeneration (Schultz et al., 2003; McKay et al., 2004; Thompson et al., 2007; Pras et al., 2015; Srivastava and Tiwari, 2016), myocardial remodeling and ventricular noncompaction (Chowdhury et al., 2014; Zhou et al., 2018), diabetic nephropathy (Kim et al., 2010; Toffoli et al., 2018) and insulinoma (Naba, et al., 2017). This protein functions in diverse cell behaviors, particularly in cell adhesion between cells and basement membrane (Vogel and Hedgecock, 2001; Morrissey et al., 2014) and is involved in cell cross talk (Chowdhury et al., 2017). Consistent with previous studies (Vogel and Hedgecock, 2001; Chowdhury et al., 2014; Chowdhury et al., 2017), the differentiation process of hDPCs expressing low levels of HMCN1 showed decreased migration ability. We hypothesized that the defective cell migration is due to the low adhesion ability of HMCN1. In *C. elegans*, hemicentin, plakin, and integrin form B-LINK, an adhesion system that links tissues by connecting adjacent basement membranes. For the highly conserved structure of HMCN1, it is reasonable to hypothesize that, in human dental root formation, HMCN1 is involved in a similar adhesion system and may lead to the migration of hDPCs to the right position—the margin of dental pulp—to differentiate into functional odontoblasts.

Additionally, the typical temporospatial expression of Hmcn1 in the teeth of PN mice intrigued us more for its distribution around cells differentiating into odontoblasts and mature odontoblasts on the root-forming side, peaking at the starting point of root formation. This kind of temporospatial expression pattern was a clue that HMCN1 might play a positive role in mesenchymal stem cell differentiation. It is known that uneven distribution of the ECM could influence the asymmetrical division of stem cells (Yennek, et al., 2014), and signal transduction cascades amplify the initial signal when minor changes in the upstream components could result in absolutely different outcomes. Since the involvement of HMCN1 in adhesion and its ability to regulate the TGF-β signaling pathway have been proven (Morrissey et al., 2014; Chowdhury et al., 2017), it is suggested that the asymmetrical distribution of HMCN1 results in asymmetrical division of dental stem cells by modulating the cell adhesion system and influencing the signal intensity. However, a recent study of stem cells in worms suggested that hemicentin restricted the stem cell compartment (Lindsay-Mosher, et al., 2020). This is possibly the result of different reactions of distinct types of cells to TGF-β and other cytokines. It has been documented that tooth ECM released ample amounts of growth factors after conditioning, including TGF-β, BMP2, VEGF, and basic fibroblast growth factor, which activate key signaling pathways. TGF-β signaling involves in the root development, when interactions between the dental mesenchyme and HERS contribute to odontoblastic and periodontal differentiation (Sui et al., 2023). We hypothesized that HMCN1 regulated root dentin formation possibly by modulating the TGF signaling pathway. In addition, mechanotransductive pathways play an important role in ECM regulation of cell behavior. HMCN1 markedly regulate cancer associated fibroblasts migration via the RhoA/ROCK signaling pathway (Liu et al., 2019). The RhoA/ROCK signaling pathway is central to mechanotransduction as it plays a key role in regulating the actin cytoskeleton and responsing to mechanical forces (Xie et al., 2023). It has been demonstrated that RhoA/ROCK signaling pathway have a regulatory role in Runx2 expression in hDPSCs during dentinogenesis (Huang H. et al., 2018). HMCN1 possibly regulated dentinogenesis through the RhoA/ROCK signaling pathway.

In summary, we identified several genes that have significantly different expression levels between the lingual and labial sides of mouse incisors and selected *Hmcn1* as our target gene for further study. Hemicentin-1, as the product of *Hmcn1*, has been proven to play an important role in the early stage of the odontogenic differentiation process of mesenchymal stem cells *in vivo*, and this ECM protein could promote mineralization and odontogenic differentiation of hDPCs *in vitro*. In addition, we speculated that HMCN1 could also enhance dental stem cell differentiation by increasing the transduction of extracellular signals like TGF-β1 mentioned in the previous study (Chowdhury et al., 2017), which should be further investigated in future research. Above all, the potential of HMCN1 in tooth root regeneration is promising, and this protein still needs to be tested *in vivo* to determine if its induction ability of root dentin formation is satisfied. In this study, we have only verified the role of HMCN1 on root development by knocking down HMCN1 in hDPCs preliminarily. For further research, HMCN1 recombinant protein will be added into the medium to mimic the gain of function of Hmcn1 *in vitro*. Further experiments should be carried out *in vivo* to prove HMCN1 plays an important role in tooth root development by conditional knockout of HMCN1 in odontoblasts of mice.

## 5 Conclusion

*Hmcn1* is an important gene in odontogenesis, especially in root dentin formation. The protein product HMCN1, an extracellular matrix protein, could regulate hDPCs odontogenic differentiation and migration. Thus, HMCN1 is an important regulator in tooth root development and could be a potential target in tooth root repair or regeneration.

## Data Availability

The original contributions presented in the study are included in the article/Supplementary Material, further inquiries can be directed to the corresponding author.

## Supplementary material

SUPPLEMENTARY FIGURE S1 The expression of HMCN1 in the epithelial tissues around the incisor cervical loop.

## References

[B1] Bonacossa-Pereira I. Coakley S. Hilliard M. A. (2022). Neuron-epidermal attachment protects hyper-fragile axons from mechanical strain. Cell Rep. 38 (10), 110501. 10.1016/j.celrep.2022.110501 35263583

[B2] Bosshardt D. D. Nanci A. (1997). Immunodetection of enamel- and cementum-related (bone) proteins at the enamel-free area and cervical portion of the tooth in rat molars. J. Bone Min. Res. 12 (3), 367–379. 10.1359/jbmr.1997.12.3.367 9076579

[B3] Chowdhury A. Hasselbach L. Echtermeyer F. Jyotsana N. Theilmeier G. Herzog C. (2017). Fibulin-6 regulates pro-fibrotic TGF-β responses in neonatal mouse ventricular cardiac fibroblasts. Sci. Rep. 7, 42725. 10.1038/srep42725 28209981 PMC5314373

[B4] Chowdhury A. Herzog C. Hasselbach L. Khouzani H. L. Zhang J. Hammerschmidt M. (2014). Expression of fibulin-6 in failing hearts and its role for cardiac fibroblast migration. Cardiovasc. Res. 103 (4), 509–520. 10.1093/cvr/cvu161 24951538

[B5] Cote L. E. Simental E. Reddien P. W. (2019). Muscle functions as a connective tissue and source of extracellular matrix in planarians. Nat. Commun. 10 (1), 1592. 10.1038/s41467-019-09539-6 30962434 PMC6453901

[B6] Cui Y. Xie J. Cai L. Zhang D. Sun J. Zhou X. (2023). Berberine regulates bone metabolism in apical periodontitis by remodelling the extracellular matrix. Oral Dis. 29 (3), 1184–1196. 10.1111/odi.14094 34874590

[B7] Cui Y. Xie J. Fu Y. Li C. Zheng L. Huang D. (2020). Berberine mediates root remodeling in an immature tooth with apical periodontitis by regulating stem cells from apical papilla differentiation. Int. J. Oral Sci. 12 (1), 18. 10.1038/s41368-020-0085-7 32555173 PMC7300019

[B8] de Vega S. Iwamoto T. Yamada Y. (2009). Fibulins: multiple roles in matrix structures and tissue functions. Cell. Mol. Life Sci. CMLS 66 (11-12), 1890–1902. 10.1007/s00018-009-8632-6 19189051 PMC11115505

[B9] Grada A. Otero-Vinas M. Prieto-Castrillo F. Obagi Z. Falanga V. (2017). Research techniques made simple: analysis of collective cell migration using the wound healing assay. J. Invest. Dermatol. 137 (2), e11–e16. 10.1016/j.jid.2016.11.020 28110712

[B10] Huang H. Wang J. Zhang Y. Zhu G. Li Y. P. Ping J. (2018a). Bone resorption deficiency affects tooth root development in RANKL mutant mice due to attenuated IGF-1 signaling in radicular odontoblasts. Bone 114, 161–171. 10.1016/j.bone.2017.12.026 29292230

[B11] Huang X. Chen X. Chen H. Xu D. Lin C. Peng B. (2018b). Rho/Rho-associated protein kinase signaling pathway-mediated downregulation of runt-related transcription factor 2 expression promotes the differentiation of dental pulp stem cells into odontoblasts. Exp. Ther. Med. 15 (5), 4457–4464. 10.3892/etm.2018.5982 29731830 PMC5920824

[B12] Huang X. Xu X. Bringas P. Hung Y. P. Chai Y. (2010). Smad4-Shh-Nfic signaling cascade-mediated epithelial-mesenchymal interaction is crucial in regulating tooth root development. J. Bone Min. Res. 25 (5), 1167–1178. 10.1359/jbmr.091103 PMC3153373 19888897

[B13] Jernvall J. Thesleff I. (2000). Reiterative signaling and patterning during mammalian tooth morphogenesis. Mech. Dev. 92 (1), 19–29. 10.1016/s0925-4773(99)00322-6 10704885

[B14] Jiang L. Sun J. Huang D. (2022). Role of slit/robo signaling pathway in bone metabolism. Int. J. Biol. Sci. 18 (3), 1303–1312. 10.7150/ijbs.66931 35173554 PMC8771833

[B15] Jing J. Feng J. Yuan Y. Guo T. Lei J. Pei F. (2022). Spatiotemporal single-cell regulatory atlas reveals neural crest lineage diversification and cellular function during tooth morphogenesis. Nat. Commun. 13 (1), 4803. 10.1038/s41467-022-32490-y 35974052 PMC9381504

[B16] Jordan S. N. Olson S. Canman J. C. (2011). Cytokinesis: thinking outside the cell. Curr. Biol. 21 (3), R119–R121. 10.1016/j.cub.2010.12.040 21300276

[B17] Kim S. Abboud H. E. Pahl M. V. Tayek J. Snyder S. Tamkin J. (2010). Examination of association with candidate genes for diabetic nephropathy in a Mexican American population. Clin. J. Am. Soc. Nephrol. 5 (6), 1072–1078. 10.2215/CJN.06550909 20299368 PMC2879299

[B18] Li L. Saiyin W. Zhang H. Wang S. Xu Q. Qin C. (2019). FAM20A is essential for amelogenesis, but is dispensable for dentinogenesis. J. Mol. Histol. 50 (6), 581–591. 10.1007/s10735-019-09851-x 31667691 PMC6861166

[B19] Lindsay-Mosher N. Chan A. Pearson B. J. (2020). Planarian EGF repeat-containing genes megf6 and hemicentin are required to restrict the stem cell compartment. PLoS Genet. 16 (2), e1008613. 10.1371/journal.pgen.1008613 32078629 PMC7059952

[B20] Liu C. L. Pan H. W. Torng P. L. Fan M. H. Mao T. L. (2019). SRPX and HMCN1 regulate cancer-associated fibroblasts to promote the invasiveness of ovarian carcinoma. Oncol. Rep. 42 (6), 2706–2715. 10.3892/or.2019.7379 31638245

[B21] Liu Y. Feng J. Li J. Zhao H. Ho T. V. Chai Y. (2015). An Nfic-hedgehog signaling cascade regulates tooth root development. Development 142 (19), 3374–3382. 10.1242/dev.127068 26293299 PMC4631759

[B22] McKay G. J. Clarke S. Hughes A. McConnell V. Schultz D. W. Klein M. L. (2004). A novel diagnostic test detects a low frequency of the hemicentin Gln5345Arg variant among Northern Irish age related macular degeneration patients. Mol. Vis. 10, 682–687. 15467524

[B23] Morrissey M. A. Keeley D. P. Hagedorn E. J. McClatchey S. T. H. Chi Q. Hall D. H. (2014). B-LINK: a hemicentin, plakin, and integrin-dependent adhesion system that links tissues by connecting adjacent basement membranes. Dev. Cell 31 (3), 319–331. 10.1016/j.devcel.2014.08.024 25443298 PMC4254419

[B24] Morsczeck C. (2022). Mechanisms during osteogenic differentiation in human dental follicle cells. Int. J. Mol. Sci. 23 (11), 5945. 10.3390/ijms23115945 35682637 PMC9180518

[B25] Naba A. Clauser K. R. Mani D. R. Carr S. A. Hynes R. O. (2017). Quantitative proteomic profiling of the extracellular matrix of pancreatic islets during the angiogenic switch and insulinoma progression. Sci. Rep. 7, 40495. 10.1038/srep40495 28071719 PMC5223159

[B26] Novoseletskaya E. S. Evdokimov P. V. Efimenko A. Y. (2023). Extracellular matrix-induced signaling pathways in mesenchymal stem/stromal cells. Cell Commun. Signal. 21 (1), 244. 10.1186/s12964-023-01252-8 37726815 PMC10507829

[B27] Pemberton T. J. Li F. Y. Oka S. Mendoza-Fandino G. A. Hsu Y. H. Bringas P. Jr (2007). Identification of novel genes expressed during mouse tooth development by microarray gene expression analysis. Dev. Dyn. 236 (8), 2245–2257. 10.1002/dvdy.21226 17626284 PMC4457363

[B28] Pras E. Kristal D. Shoshany N. Volodarsky D. Vulih I. Celniker G. (2015). Rare genetic variants in Tunisian Jewish patients suffering from age-related macular degeneration. J. Med. Genet. 52 (7), 484–492. 10.1136/jmedgenet-2015-103130 25986072

[B29] Schultz D. W. Klein M. L. Humpert A. J. Luzier C. W. Persun V. Schain M. (2003). Analysis of the ARMD1 locus: evidence that a mutation in HEMICENTIN-1 is associated with age-related macular degeneration in a large family. Hum. Mol. Genet. 12 (24), 3315–3323. 10.1093/hmg/ddg348 14570714

[B30] Shin Y. K. Cheon S. Kim S. D. Moon J. S. Kim J. Y. Kim S. H. (2021). Identification of novel candidate genes implicated in odontogenic potential in the developing mouse tooth germ using transcriptome analysis. Genes Genom 43 (9), 1087–1094. 10.1007/s13258-021-01130-y 34302633

[B31] Sisto M. D'Amore M. Lofrumento D. D. Scagliusi P. D'Amore S. Mitolo V. (2009). Fibulin-6 expression and anoikis in human salivary gland epithelial cells: implications in Sjogren's syndrome. Int. Immunol. 21 (3), 303–311. 10.1093/intimm/dxp001 19190085

[B32] Srivastava P. Tiwari A. (2016). A new insight of herbal promises against ocular disorders: an occuloinformatics approach. Curr. Top. Med. Chem. 16 (6), 634–654. 10.2174/1568026615666150819105716 26286213

[B33] Sriwattanapong K. Theerapanon T. Khamwachirapitak C. Sae-Ear P. Sa-Ard-Iam N. Shotelersuk V. (2024). In-depth investigation of FAM20A insufficiency effects on deciduous dental pulp cells: altered behaviours, osteogenic differentiation, and inflammatory gene expression. Int. Endod. J. 57 (6), 745–758. 10.1111/iej.14056 38477421

[B34] Sui B. D. Zheng C. X. Zhao W. M. Xuan K. Li B. Jin Y. (2023). Mesenchymal condensation in tooth development and regeneration: a focus on translational aspects of organogenesis. Physiol. Rev. 103 (3), 1899–1964. 10.1152/physrev.00019.2022 36656056

[B35] Sun J. X. Horst O. V. Bumgarner R. Lakely B. Somerman M. J. Zhang H. (2012). Laser capture microdissection enables cellular and molecular studies of tooth root development. Int. J. Oral Sci. 4 (1), 7–13. 10.1038/ijos.2012.15 22422086 PMC3412663

[B36] Thesleff I. Sharpe P. (1997). Signalling networks regulating dental development. Mech. Dev. 67 (2), 111–123. 10.1016/s0925-4773(97)00115-9 9392510

[B37] Thompson C. L. Klein B. E. Klein R. Xu Z. Capriotti J. Joshi T. (2007). Complement factor H and hemicentin-1 in age-related macular degeneration and renal phenotypes. Hum. Mol. Genet. 16 (17), 2135–2148. 10.1093/hmg/ddm164 17591627

[B38] Toffoli B. Zennaro C. Winkler C. Giordano Attianese G. M. P. Bernardi S. Carraro M. (2018). Hemicentin 1 influences podocyte dynamic changes in glomerular diseases. Am. J. Physiol.-Renal Physiol. 314 (6), F1154–F1165. 10.1152/ajprenal.00198.2017 29488390

[B39] Tucker A. Sharpe P. (2004). The cutting-edge of mammalian development; how the embryo makes teeth. Nat. Rev. Genet. 5 (7), 499–508. 10.1038/nrg1380 15211352

[B40] Vogel B. E. Hedgecock E. M. (2001). Hemicentin, a conserved extracellular member of the immunoglobulin superfamily, organizes epithelial and other cell attachments into oriented line-shaped junctions. Development 128 (6), 883–894. 10.1242/dev.128.6.883 11222143

[B41] Welcker D. Stein C. Feitosa N. M. Armistead J. Zhang J. L. Lütke S. (2021). Hemicentin-1 is an essential extracellular matrix component of the dermal-epidermal and myotendinous junctions. Sci. Rep. 11 (1), 17926. 10.1038/s41598-021-96824-4 34504132 PMC8429575

[B42] Xie N. Xiao C. Shu Q. Cheng B. Wang Z. Xue R. (2023). Cell response to mechanical microenvironment cues via Rho signaling: from mechanobiology to mechanomedicine. Acta Biomater. 159, 1–20. 10.1016/j.actbio.2023.01.039 36717048

[B43] Yennek S. Burute M. Théry M. Tajbakhsh S. (2014). Cell adhesion geometry regulates non-random DNA segregation and asymmetric cell fates in mouse skeletal muscle stem cells. Cell Rep. 7 (4), 961–970. 10.1016/j.celrep.2014.04.016 24836002

[B47] Yoshizaki K. Yamada Y. (2013). Gene evolution and functions of extracellular matrix proteins in teeth. Orthod. Waves 72 (1), 1–10. 10.1016/j.odw.2013.01.040 23539364 PMC3607546

[B44] Yu T. Klein O. D. (2020). Molecular and cellular mechanisms of tooth development, homeostasis and repair. Development 147 (2), dev184754. 10.1242/dev.184754 31980484 PMC6983727

[B45] Zhang Y. D. Chen Z. Song Y. Q. Liu C. Chen Y. P. (2005). Making a tooth: growth factors, transcription factors, and stem cells. Cell Res. 15 (5), 301–316. 10.1038/sj.cr.7290299 15916718

[B46] Zhou Y. Qian Z. Yang J. Zhu M. Hou X. Wang Y. (2018). Whole exome sequencing identifies novel candidate mutations in a Chinese family with left ventricular noncompaction. Mol. Med. Rep. 17 (5), 7325–7330. 10.3892/mmr.2018.8777 29568952

