# Supplementary figures and images for: Hemicentin-1 is an essential extracellular matrix component during tooth root formation by promoting mesenchymal cells differentiation
Source: Front Cell Dev Biol. 2024 Jul 10;12:1435241. doi: 10.3389/fcell.2024.1435241 (PMC11266140; doi:10.3389/fcell.2024.1435241)

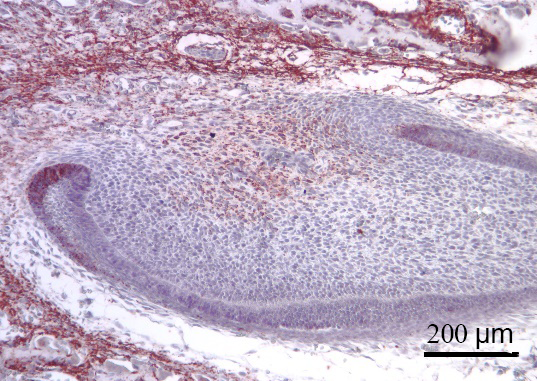

Supplement: Supplementary file 1 [file Image1.TIF]
